# The Healthy Men Study: An Evaluation of Exposure to Disinfection By-Products in Tap Water and Sperm Quality

**DOI:** 10.1289/ehp.10120

**Published:** 2007-05-22

**Authors:** Thomas J. Luben, Andrew F. Olshan, Amy H. Herring, Susan Jeffay, Lillian Strader, Rebecca M. Buus, Ronna L. Chan, David A. Savitz, Philip C. Singer, Howard S. Weinberg, Sally D. Perreault

**Affiliations:** 1 Department of Epidemiology and; 2 Department of Biostatistics, University of North Carolina School of Public Health, Chapel Hill, North Carolina, USA; 3 Reproductive Toxicology Division, Office of Research and Development, U.S. Environmental Protection Agency, Research Triangle Park, North Carolina, USA; 4 Department of Community and Preventive Medicine, Mount Sinai School of Medicine, New York, New York, USA; 5 Department of Environmental Sciences and Engineering, University of North Carolina School of Public Health, Chapel Hill, North Carolina USA

**Keywords:** disinfection by-products, environmental exposure, epidemiology, haloacetic acids, human, male, reproduction, semen, sperm count, trihalomethanes

## Abstract

**Background:**

Chlorination of drinking water generates disinfection by-products (DBPs), which have been shown to disrupt spermatogenesis in rodents at high doses, suggesting that DBPs could pose a reproductive risk to men. In this study we assessed DBP exposure and testicular toxicity, as evidenced by altered semen quality.

**Methods:**

We conducted a cohort study to evaluate semen quality in men with well-characterized exposures to DBPs. Participants were 228 presumed fertile men with different DBP profiles. They completed a telephone interview about demographics, health history, water consumption, and other exposures and provided a semen sample. Semen outcomes included sperm concentration and morphology, as well as DNA integrity and chromatin maturity. Exposures to DBPs were evaluated by incorporating data on water consumption and bathing and showering with concentrations measured in tap water. We used multivariable linear regression to assess the relationship between exposure to DBPs and adverse sperm outcomes.

**Results:**

The mean (median) sperm concentration and sperm count were 114.2 (90.5) million/mL and 362 (265) million, respectively. The mean (median) of the four trihalomethane species (THM4) exposure was 45.7 (65.3) μg/L, and the mean (median) of the nine haloacetic acid species (HAA9) exposure was 30.7 (44.2) μg/L. These sperm parameters were not associated with exposure to these classes of DBPs. For other sperm outcomes, we found no consistent pattern of increased abnormal semen quality with elevated exposure to trihalomethanes (THMs) or haloacetic acids (HAAs). The use of alternate methods for assessing exposure to DBPs and site-specific analyses did not change these results.

**Conclusions:**

The results of this study do not support an association between exposure to levels of DBPs near or below regulatory limits and adverse sperm outcomes in humans.

Disinfection by-products (DBPs) form when chlorine or other disinfectants react with organic matter during preparation of drinking water. There are many classes of DBPs, including trihalomethanes (THMs) and haloacetic acids (HAAs). The relative concentrations of these DBPs, as well as the proportional distributions of individual chemicals within these classes, vary based on source water characteristics and treatment methods and on distribution system characteristics. The U.S. Environmental Protection Agency (EPA) and others have evaluated the potential adverse health effects of DBPs in both toxicologic and epidemiologic research. Several epidemiologic studies have suggested a possible association between DBPs and adverse pregnancy outcomes (Klotz and Pyrch 1999; Savitz et al. 1995, 2005, 2006; Toledano et al. 2005; Waller et al. 1998; Wright et al. 2003). To date, only one study has addressed DBPs and male reproductive health wherein exposure to DBPs was associated with decrements in sperm motility (Fenster et al. 2003).

Animal studies have consistently demonstrated an association between oral exposure to HAAs and adverse effects in the male reproductive system (Christian et al. 2002; Linder et al. 1994a, 1994b, 1995, 1997a, 1997b; Veeramachaneni et al. 2000). Testicular toxicity, including acute spermatotoxicity (Linder et al. 1994b), impaired reproductive competence and sperm quality (Linder et al. 1995), delayed spermiation and distorted sperm motility and morphology (Linder et al. 1997a), histopathologic changes in testis and epididymis (Linder et al. 1997b), transient subfertility (Luft et al. 2000), and altered sperm production and epididymal tubule changes (Christian et al. 2002), has been demonstrated when rodents were exposed to high doses (in the milligrams per kilogram range) of selected individual HAA species, with dibromoacetic acid being the most studied.

These rodent toxicology studies suggest that HAAs in drinking water, especially the brominated species, could pose a risk to the male reproductive system in humans, and that an evaluation of similar outcomes in human semen (sperm numbers, morphology, and motility) would be appropriate. Continued or intermittent exposure of men to DBPs in drinking water, even at levels below those that are acutely toxic to rodents, may have the potential to produce testicular toxicity as evidenced by altered semen quality.

We conducted a cohort study to address this possibility by evaluating semen quality in men with well-characterized exposures to DBPs. We were able to integrate this study with a cohort study of drinking-water DBPs and pregnancy loss (Savitz et al. 2006). Recruitment for both studies involved couples in three geographic locations in the United States, with our study adding home semen collection in a subcohort. We examined exposure to THMs and HAAs based on the concentration of these two classes of DBPs (as well as the four individual species of THMs and nine individual species of HAAs) and to TOX (total organic halides) measured weekly in the tap water from distribution systems serving the three study sites. These study sites were selected specifically to provide a reference site with low overall DBPs and two sites with relatively high levels of DPBs but differing with respect to brominated versus chlorinated species. Based on the concentrations of four regulated THMs (THM4), nine HAAs (HAA9), and total organic halides (TOX) measured weekly in tap water sampled from the distribution systems (Savitz et al. 2006), we calculated mean levels of DBPs over the 90 days preceding semen sampling. We then used questionnaire data to estimate the daily volume of tap water ingested and the frequency and duration of bathing and showering for each participant; we used these data to create exposure indices estimating exposure to DBPs via the ingestion, inhalation, and dermal routes of exposure. In the statistical analyses, we considered exposure to combined DBPs in accordance with current regulations [i.e., THM4 and HAA5 (sum of five regulated haloacetic acids)], as well as HAA9, to capture maximum exposures to HAAs. Because brominated HAAs may be more potent male reproductive toxicants, we also considered the composite concentration of brominated HAAs (HAA-Br) and THMs (THM-Br).

## Methods

### Study design and subject recruitment

The basic study design of this study, “The Healthy Men Study” (HMS), has been described previously (Olshan et al. 2007). The University of North Carolina School of Public Health’s Institutional Review Board approved the study protocol, and all study participants gave written informed consent. Briefly, the HMS identified male partners of women who participated in a prospective study of drinking-water DBPs and spontaneous abortion (the “Right From the Start” (RFTS) study (Savitz et al. 2005, 2006). Men were prospectively identified from the RFTS study and recruited from the three RFTS study sites. The sampling strategy included one group of men from a study site with very low levels of all targeted DBPs (low-DBP site), one group from a site with moderate levels of chlorinated DBPs and lower levels of brominated DBPs (chlorinated DBP site), and one group from a site with moderate levels of brominated DBPs and lower levels of chlorine-containing species (brominated DBP site). The term “moderate” is used here to describe exposures that approach but are still below the limits established by the U.S. EPA for regulated DBPs (i.e., 80 μg/L THM4 and 60 μg/L HAA5) (U.S. EPA 1998).

### Questionnaire

A computer-assisted telephone interview was administered to each participant by experienced interviewers, with responses entered directly into a computerized database. The average duration of the interview was approximately 40 min. Questions covered the following topics: general lifestyle, health, reproductive history, environment, diet, stress, occupational exposures, hobbies, and demographic factors, as well as drinking-water consumption and water exposures. The section of the interview regarding drinking-water consumption and water exposures included questions about the quantity (volume) of hot and cold drinks consumed at home and at work, use of household water filters and filter types, frequency and duration of bathing and showering, and frequency and duration of pool and Jacuzzi use. Participants were provided with a diagram of small, medium, and large glasses, with the relative volume of each listed in cups, pints, quarts, and ounces to facilitate the accurate estimation of daily personal consumption at home and at work. Participants also gave the street address of their home, workplace, and other locations where they spent a significant amount of time in order for us to determine if these locations were within the water utility service area.

### Semen collection and analyses

Participants were asked to provide a single semen sample using a special kit designed to allow the man to collect a semen specimen in the privacy of his own home and at a time convenient to him (Royster et al. 2000). Before sending the kit to the participant, study staff confirmed by telephone the participant’s mailing address, gave brief instructions on how to use the kit, and asked the participant if he had experience doing a similar collection in the past. Verbal instructions included the importance of doing the collection after 2–7 days of abstinence from sexual activity. The kit was mailed to the participant during the week before his anticipated collection date. Participants in the low DBP site and the brominated DBP site were instructed to open the box as soon as they received it because the package mailed to those sites also contained ice packs that would need to be frozen at least 24 hr before collection and packaged with the sample before mailing.

The instructions accompanying the kit included photographs and instructions on how to properly collect the specimen, package and prepare it for shipping, and call to arrange the courier pickup. Because the package was delivered to the laboratory by overnight carrier for residents of the low- and brominated-DBP sites, participants from these sites were instructed to collect the specimen only on Monday, Tuesday, or Wednesday, between 0500 and 1700 hours. Participants in the chlorinated DBP site were told to collect the specimen between 0800 and 1200 hours so the courier could pick up the package and deliver it to the U.S. EPA laboratory within 1.5 hr of collection. Upon receipt of samples from the chlorinated DBP site, aliquots were removed for immediate processing, and the remainder was stored overnight in the refrigerator to mimic overnight shipping. A second set of aliquots was processed the next morning for comparison with the fresh sample.

When the initial specimen volume was very low (< 0.5 mL), the man reported spillage or incomplete sample collection, shipping was delayed or the sample was not packaged correctly, or the participant’s abstinence interval was too far out of the suggested 2–7 day range, participants were asked to provide a second or third specimen. This affected 10% (*n* = 20) of participants, of which all 20 complied with the repeat collections.

All samples were received by a single laboratory at the U.S. EPA. Immediately upon receipt, semen volume was measured, and aliquots were removed for determination of sperm concentration by IVOS-IDENT (Hamilton Thorne Research, Beverly, MA; Zinaman et al. 1996), from which total sperm count was calculated. Additional aliquots were used to prepare smears that were air-dried and stored for later analyses of sperm morphology [World Health Organization (WHO) 1999]. Sperm motility, which declines over time and is therefore not a reliable measure for shipped semen, was not included in the statistical analysis. However, sperm motility and viability (using propitium iodide as a vital stain) were monitored. All samples retained motile and viable sperm, an indication that the sample had been collected and shipped according to instructions.

Additional aliquots (0.1 mL) were frozen and stored at –70°C for later analysis of chromatin integrity by the sperm chromatin structure assay (SCSA; Evenson and Jost 2000) and for chromatin maturity by chromomycin A3 (CMA) staining (Sakkas et al. 1995). These measures were included because some DBPs are considered to be carcinogenic and might, therefore, damage sperm DNA. For the SCSA, aliquots were shipped on liquid nitrogen to SCSA Diagnostics (Brookings, SD) for analysis according to established methods (Evenson et al. 2002). Briefly, the sample is exposed to an acid buffer (pH 1.2) for 30 sec to denature abnormal chromatin/DNA; the sperm is then stained with acridine orange, a metachromatic dye that fluoresces red when bound to denatured (fragmented) DNA and green when bound to intact DNA. Fluorescence is measured by flow cytometry in each of 5,000 sperm per sample. SCSA software is used to calculate the percentage of sperm with fragmented DNA (DNA fragmentation; DFI). Percent DFI has been shown to correlate with other tests of sperm DNA damage such as TUNEL and COMET assays.

The CMA assay is based on the stainability of sperm with CMA3. This fluorescent DNA dye intercalates into DNA and stains nuclei green. However, it cannot bind sperm chromatin in which protamine has replaced histone. Hence, sperm that stain with CMA3 are considered “underprotamined” or immature (Sakkas et al. 1995). Aliquots of frozen sperm were later thawed in groups, stained with CMA3, and scored visually. Sperm were considered CMA3 positive when at least 50% of the area of the nucleus fluoresced above background. Clinical studies have shown an association between relatively high percentages of CMA3 staining and sub/infertility (Bianchi et al. 1993; Esterhuizen et al. 2000). In both SSA and CMA assays, aliquots of pooled semen were included in each run to serve as an internal standard.

In this article we report on nine sperm outcomes reflective of testis function: sperm count (million), sperm concentration (million per milliliter), sperm morphology (percent normal sperm) and its components (percent of sperm cells with abnormal head, percent of sperm cells with abnormal midsection, percent of sperm cells with abnormal tails, and percent of sperm cells with abnormal cytoplasmic drop), percent sperm with DNA fragmentation according to SCSA (percent DFI), and percent immature sperm according to CMA staining.

### Disinfection by-product data and exposure categories

Our investigation made use of exposure data collected for the RFTS study. The water sampling and analytic methodologies from RFTS have been described previously (Savitz et al. 2005, 2006). Water was sampled at frequent intervals (weekly or biweekly) at a representative location in each of the three study sites and analyzed for THM4, HAA9, and TOX. Samples were periodically collected at several points in the distribution system to verify that the sampling locations chosen had THM, HAA, and TOX concentrations that were indeed representative of the entire system on that day. Because the brominated DBP site and the chlorinated DBP site both used combined chlorine as a terminal disinfectant, no spatial variabiltity in DBP concentrations was observed on any given day. Sample collection was performed by field personnel in accordance with a specified protocol. Residual chlorine concentrations and temperature were also measured at the time of DBP sample collection. Samples were collected near midday from a cold water tap that had been run for at least 5 min before sample collection. Weekly tap water samples were collected from the chlorinated DBP between 10 October 2000 and 29 February 2004. Biweekly tap water samples were collected from the low DBP site between 30 July 2001 and 1 August 2004. Weekly tap water samples were collected from the brominated DBP site between 3 June 2002 and 5 September 2004. These sampling periods included the 90 days before semen samples were collected, such that each man could be assigned an exposure value based on these data.

We chose to estimate exposures to DBPs in three ways: *a*) the DBP concentration measured in the subject’s tap water averaged for the 90 days before semen sampling; *b*) the product of the measured DBP concentration and volume of water ingested by each participant, adjusting for water filtration and boiling in the preparation of hot drinks; and *c*) the measured DBP concentration and time spent bathing and showering. The simplest level of analysis defined exposure based solely on the weekly concentrations of DBPs in tap water in the distribution system serving the man’s home, as measured for the purposes of the study. The next method of exposure assignment incorporated information on ingestion of tap water and the major influences on DBP concentration in ingested tap water, namely point-of-use filters and heating. The volume of water consumed was multiplied by the tap water concentration and, after adjustment for filter use and heating, presented as an estimated dose with units of micrograms per day (Savitz et al. 2005, 2006). Because DBP levels in tap water may be altered if the consumer uses a filter or boils water before ingestion, it was necessary to adjust the tap water concentrations to take these factors into account. Due to these modifications, assignment of DBP levels via ingestion was calculated using a series of adjustment factors and equations based on the findings from a previous study (Savitz et al. 2005). Finally, we examined information on bathing and showering to estimate exposure to THMs, which are volatilized and inhaled as well as being absorbed dermally. The duration of showering and bathing, combined with knowledge of the DBP concentrations in the tap water, was used to estimate absorbed and inhaled amounts based on previous literature (Savitz et al. 2006). We considered but ultimately chose not to use an integrated exposure classification that incorporated tap water concentration, ingestion, and bathing and showering behaviors due to the high correlation between this integrated exposure classification and the isolated bathing and showering classification (*r* = 0.97). In addition, ingested THMs are rapidly metabolized by the liver, whereas inhaled or dermally absorbed THMs are not (Haddad et al. 2006). Thus, addressing bathing and showering exposures alone assesses the effect of unmetabolized THMs potentially affecting reproductive health outcomes.

Assignment of DBP concentrations for each man required information on the timing of exposure and the array of week-specific DBP measurements. For each DBP species, the average value for the 90 days preceding each semen sample was calculated in order to represent the average concentration to which those sperm had been exposed during their development from stem cells, through meiosis, spermiogenesis, and epididymal transit (Clermont 1963; Heller and Clermont 1964). We also conducted analyses for which the average DBP value for the 30 days and 10 days preceding each semen sample was calculated. Results from these analyses did not differ materially from those with the 90-day window, and are not presented.

The basis for selecting individual DBP species and groupings for analysis was guided by several considerations. First, evidence from toxicologic studies regarding potential reproductive toxicity was considered, encouraging evaluation of the agents most likely to be directly responsible for adverse reproductive effects, should any be found. Second, previous epidemiologic studies were considered. Although these studies mainly evaluated effects in pregnant women, not men, we followed leads that they suggested and more generally addressed a number of the same agents as others had studied. Third, the availability of monitoring data and the status of DBP regulations was considered. Based on these criteria, we chose to evaluate all individual THM and HAA species, as well as THM4, HAA5, HAA9, the sum of all brominated THMs and HAAs (THM-Br and HAA-Br, respectively), and TOX. THM4 represents the sum of the concentrations of bromoform, chloroform, bromodichloromethane, and dibromochloromethane; HAA5 represents the sum of the concentrations of monobromoacetic acid, monochloroacetic acid, dibromoacetic acid, dichloroacetic acid, and trichloroacetic acid. HAA9 represents the sum of the concentrations of HAA5 plus bromochloroacetic acid, bromodichloroacetic acid, dibromochloroacetic acid, and tribromoacetic acid. TOX represents the sum of all measured DBPs plus other halogenated organics of unknown identity.

### Statistical analyses

We performed statistical transformations on several of the outcome variables to better approximate the normality assumption of the linear model. Specifically, a natural log transformation was applied to the sperm count and concentration variables, and an arc sine transformation was applied to the percent normal sperm cells, percent of sperm cells with abnormal head, percent of sperm cells with abnormal midsection, percent of sperm cells with abnormal tails, and percent of sperm cells with abnormal cytoplasmic drop. For interpretability, each of the outcome variables was standardized (after statistical transformation, if applied) such that the SD and the variance were equal to one. Thus each beta coefficient provides an estimate of effect in terms of a change in SDs of the response variable.

We characterized the distribution of demographic, exposure, and other characteristics for all participants and by individual study site. In addition, we conducted bivariate analyses for all covariates and exposure variables with each of the outcome variables. Linear regression was used to assess the association between each exposure variable and each outcome, adjusted for potential confounders. We entered all potential covariates into a linear regression model (one model for each outcome) with THM4 concentration as the dichotomous exposure variable (top 50th percentile vs. bottom 50th percentile) for the exposure window 90 days before sample collection. The current U.S. EPA maximum contaminant level for THM4 in drinking water is a running annual average of 80 μg/L in samples measured quarterly (U.S. EPA 1998). Because so few of our participants had exposures exceeding this limit, and those who did exceed the limit did not do so by much, we chose to dichotomize the exposure variables and compare the top 50th exposure percentile to the bottom 50th exposure percentile in preliminary analyses. In subsequent analyses with all exposure variables, we chose to compare the top 25th and top 10th exposure percentiles to the bottom 50th exposure percentile. We evaluated potential confounders using the 10% rule, and we found that none of those evaluated [paternal age, days abstaining, body mass index (BMI), income, education, alcohol consumption, race, ethnicity, smoking, fever, infection or other illness, caffeine use, and season the sample was collected] met this definition of confounding because results did not differ by more than 10% with adjustment for covariates. We present results adjusted for paternal age, days abstaining, and education in order to facilitate comparison among previously published studies of semen quality and environmental contaminants. Because of the large number of comparisons (162), no adjustment for multiple comparisons, and the moderate correlations among outcomes and exposures of interest, associations of modest statistical significance should be cautiously interpreted.

In addition to fitting models with a dichotomous exposure variable, we calculated partial correlation coefficients for all sperm outcomes and the selected groupings of DBPs as continuous variables adjusted for paternal age, days abstaining, and education. These partial correlation coefficients describe the linear association between DBP exposure and each (transformed) sperm outcome, adjusting for age, abstinence, and education.

## Results

### Participation and description of the study group

As detailed in a preliminary report (Olshan et al. 2007), we attempted to recruit the male partners of 960 female participants from the RFTS study. Of these 960 original female participants, 154 were lost to follow up, 460 refused to participate, and 41 reported that their partner was ineligible to participate. The remaining 305 women agreed to provide their partner’s contact information. We were unable to contact 8 of these men, an additional 11 men did not meet the eligibility criteria to participate in the study, and 12 refused to participate. This left 274 (29%) men who were deemed eligible for the study. Among the 274 eligible men who were contacted, 230 completed the interview and provided an acceptable semen sample, yielding a participation of 84%. A signed informed consent form was not received from one of these participants who provided an acceptable semen sample, and this participant was excluded from analyses. One participant provided a very low volume semen sample inadequate for analysis. This participant had a vasectomy before an additional sample could be collected and was excluded from analyses. Thus, final analyses included 228 men. Selected sociodemographic characteristics for the final HMS cohort were compared with those from all men deemed eligible for the study; these results are presented in Table 1. Men participating in the study did not differ from all men eligible for the study on age, education, household income, or cigarette smoking history, but were more likely to be white and to have had more years of schooling.

Demographic characteristics and potential confounders examined for inclusion in the regression model are presented in Table 2. Men from all three sites were similar with respect to age and race. Men from the brominated DBP site were more likely to be Hispanic, less likely to have graduated from college, and had lower income than the men from the chlorinated and low DBP sites. Other potential risk factors and confounders were similar among study sites. About 40% of the participants were self-reported smokers, and > 75% reported alcohol use. None of the participants had a BMI that would classify them as underweight. No semen samples were collected from men in the chlorinated DBP site during the spring. This is important because the water utility at the chlorinated DBP site switches from combined chlorine to free chlorine for one month in the spring, leading to spatial variability in DBP exposure throughout the utility’s service area. Men would have experienced different profiles of DBP exposure had they been sampled at that time.

### Sperm quality

The mean (median) sperm concentration for the entire group was 114.23 (90.50) million/mL (Table 3) and did not differ by study site (data not shown). The current WHO reference value for sperm concentration is ≥ 20 million/mL (WHO 1999). In this group of men, < 5% had sperm concentrations < 20 million/mL. The mean (median) sperm count for the entire group was 362 (265) million.

The mean (± SD) percentage of normal sperm for all samples was 14.14 ± 5.84% (Table 3) and did not differ by study site (data not shown). The most recent WHO guidelines (WHO 1999) do not specify a reference value for this measure. Nevertheless, the guidelines note that as sperm morphology falls below 15% normal forms (using strict criteria for scoring sperm as normal), the fertilization rate *in vitro* decreases. It is notable that the mean percentage of normal sperm in our study population was just below this cutpoint for reduced fertility. The mean (± SD) percentage of abnormal sperm head, midsection, and tail were 78.59 ± 7.41%, 22.71 ± 8.89%, and 22.24 ± 14.25%, respectively. We also examined the percentage of sperm with an abnormal cytoplasmic drop. The mean (± SD) for this outcome was 1.55 ± 1.52%.

We evaluated sperm DNA and chromatin integrity by the SCSA and CMA staining. Cells with DFI values above a threshold are considered abnormal. The SCSA had a mean (± SD) percent DFI of 20 ± 12% (Table 3). This value was consistent with values reported by Wyrobek et al. (2006) in men with similar ages. The mean (± SD) CMA was 60 ± 16%, which is higher than reported by Bianchi et al. (1993) for normospermic men, possibly because our scoring criteria were more inclusive.

### Disinfection by-product data

**Figure 1:**
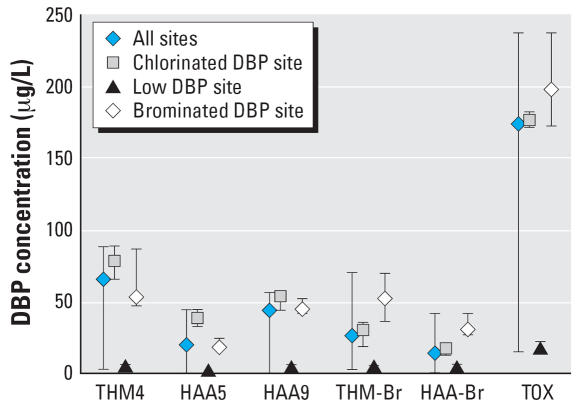
Median exposure concentrations among HMS study participants by site. Error bars indicate the range of all concentrations for each site.

The DBP concentrations for grouped THMs, HAAs, and TOX, shown in quartiles for the 90 days preceding semen sampling, are presented in Figure 1. The 90-day mean values for THM4 exceeded the upper regulatory limit of 80 μg/L for 18% of participants. The 90-day mean values for HAA5 did not exceed the upper regulatory limit of 60 μg/L for any of the participants.

DBP concentrations varied by site, as expected (Figure 1); that is, chlorinated species of THMs and HAAs were higher in the chlorinated DBP site than in the brominated DBP site. Conversely, brominated species of THMs and HAAs were higher in the brominated DBP site than the chlorinated DBP site. DBP concentrations were uniformly low in the low DBP site, as anticipated, and in many instances were below detectable levels.

The overall and site-specific exposure differences calculated using volume of tap water ingested and frequency and duration of bathing and showering data coupled with measured DBP concentration did not differ substantially from DBP concentrations presented in Figure 1. That is, exposure to chlorinated species remained highest in the chlorinated DBP site; exposure to brominated species remained highest in the brominated DBP site; and exposures remained low and nondetectable in the low DBP site (data not shown).

### Disinfection by-product exposure and sperm outcomes

Overall, our results did not reveal a consistent pattern between elevated exposure to DBPs and increased occurrence of adverse sperm outcomes. We did not find any material differences in the results of our analyses when we used algorithms to include personal consumption or bathing and showering data compared with the use of DBP concentrations in tap water alone (data not shown). We also examined different exposure windows to see if these might affect our results. Calculating exposure during the 10, 30, and 90 days before semen collection produced similar results. We present the results for exposure based on the average concentration of weekly or biweekly DBP measurements during the 90 days before semen collection. For ease of interpretation, we have presented the results for exposures to six groupings of DBPs based on measured concentrations in the distribution system: THM4, HAA5, HAA9, THM-Br, HAA-Br, and TOX. Results for individual species of THMs and HAAs are available in Supplemental Material available online (http://www.ehponline.org/docs/2007/10120/suppl.pdf). The beta coefficients from the multivariable linear regression are presented in Table 4, and the partial correlation coefficients (*r*) are shown in Table 5. Finally, we conducted site-specific analyses to see if these might affect our results (Table 5).

When interpreting the information in Tables 4 and 5, it is important to note that negative beta coefficients and correlation coefficients indicate increased adverse semen quality with elevated DBP exposure for the sperm count, sperm concentration, and percent normal sperm outcomes, whereas positive beta coefficients and correlation coefficients indicate decreased adverse semen quality with elevated DBP exposure for the percent sperm cells with abnormal heads, midsections, tails, and cytoplasmic drop; percent CMA; and percent DFI.

### Sperm count and concentration

Contrary to expectation, participants classified in the top 25th percentile of exposure to THMs had sperm counts that, on average, were one-third of an SD higher than those classified in the bottom 50th percentile of exposure [β = 0.35; 95% confidence limits (CL), −0.02, 0.72] (Table 4). Similarly, exposures to higher concentrations of HAA5 were associated with higher sperm counts when the top 25th exposure percentile was compared with the bottom 50th exposure percentile. In support of our hypothesis, increasing exposure to TOX was found to decrease sperm concentration, with participants in the top 25th percentile of TOX exposure having one-third of an SD lower sperm concentration than those in the bottom 50th percentile (β = −0.34; 95% CL, −0.66, −0.03) (Table 4).

The partial correlation coefficients in Table 5 indicate that there are no linear associations between log-transformed sperm count and sperm concentration and the six DBP groupings examined in this study.

### Sperm morphology

Again, contrary to expectation, when participants in the top 25th percentile of exposure were compared with those in the bottom 50th percentile, those with the higher exposures also had higher percentages of normal sperm cells, which was statistically significant (*p* < 0.05) (β = 0.65; 95% CL, 0.34, 0.96) (Table 4).

The beta coefficients for percent abnormal head were positive in comparisons with each of the DBP groupings, although the magnitude of each was small and none was statistically significant. Conversely, all of the coefficients for the comparisons with percent abnormal tail and DBP groupings were negative, indicating a smaller proportion of abnormal outcomes with increasing DBP exposure. These comparisons produced some of the largest coefficients in our analyses (Table 4). The coefficients for percent abnormal midsection and THM4, HAA5, and HAA9 were all negative and statistically significant (*p* < 0.05) (Table 4), indicating a smaller proportion of abnormal outcomes with increasing DBP exposure. The coefficients for comparisons made with percent abnormal midsection and THM-Br, HAA-Br, and TOX were positive but low in magnitude.

Contrary to expectations, the partial correlation coefficients in Table 5 also indicate a positive association between percent normal sperm cells and several DBP groupings. That is, the percent of normal sperm cells increased as exposure to THM4, HAA5, HAA9, and TOX increased [*r* = 0.242 (*p* < 0.001); *r* = 0.299 (*p* < 0.001); *r* = 0.245 (*p* < 0.001); and *r* = 0.184 (*p* < 0.01), respectively]. The partial correlation coefficients showed no association between exposure to any of the six DBP groupings and percent of sperm cells with abnormal head. The results for percent of sperm cells with abnormal midsection and tail did not support our hypothesis. As exposure to THM4, HAA5, HAA9, THM-Br, HAA-Br, and TOX increased, the percent of sperm cells with abnormal tails decreased (*r* = −0.498 (*p* < 0.001); *r* = −0.571 (*p* < 0.001); *r* = −0.498 (*p* < 0.001); *r* = −0.244 (*p* < 0.001); *r* = −0.200 (*p* < 0.01); and *r* = −0.413 (*p* < 0.001), respectively). Results for percent of sperm cells with abnormal midsections were similar, though the magnitudes of the coefficients were smaller.

### Abnormal cytoplasmic drop

The coefficients for percent abnormal cytoplasmic drop were positive for comparisons with THM4, HAA5, and HAA9, indicating an increase of approximately one-third of an SD with exposure to these DBP groupings [β = 0.33 (95% CL, 0.02, 0.31); β = −0.47 (95% CL, −0.02, 0.64); and β = 0.37 (95% CL, 0.04, 0.70), respectively) (Table 4).

The partial correlation coefficients show a slight positive association between percent of sperm cells with abnormal cytoplasmic drop and THM4, HAA5, and HAA9 [*r* = 0.150 (*p* < 0.05); *r* = 0.196 (*p* < 0.05); and *r* = 0.151 (*p* < 0.05), respectively].

### Sperm genetic integrity assays

Based on the results of the multivariable linear regression and the partial correlation coefficients, there was no association between percent CMA and any of the DBP groupings.

Overall, exposure to DBPs was associated with a decrease in cells with an abnormal DFI. This trend was most apparent with exposures to THM4, HAA5, and HAA9 [β = −0.41 (95% CL, −0.78, −0.05); β = −0.43 (95% CL −0.78, −0.08); and β = −0.51 (95% CL −0.85, −0.17), respectively] (Table 4). The results from the partial correlation coefficients analyses were consistent with these associations (Table 5).

## Discussion

Overall, the results of the present study do not support an association between exposure to DBPs at levels approaching regulatory limits and adverse sperm outcomes, although we did see an association between TOX and sperm concentration that was in line with our hypothesis. The results for sperm concentration are of interest with respect to our hypothesis because the association with exposure is significant for TOX, a measure of all halogenated organics in disinfected water. The lone association of TOX exposure with sperm concentration may lend support to findings that have suggested that TOX is a stronger risk factor for adverse pregnancy outcomes than any of the regulated DBP groups or species (Savitz et al. 2006) and that the toxicity of TOX is greater than that of the individual or subclasses of DBPs (Wang et al. 2007). This finding could be due, in part, to the fact that, at least for HAAs, the reproductive and developmental effects have been found to be additive across individual species (Andrews et al. 2004; Kaydos et al. 2004). Based on previous toxicology studies, we would have expected to see decrements in sperm morphology and sperm numbers after exposure to DBPs, but we did not. In fact, in toxicology studies, Linder et al. (1994a, 1994b, 1995b, 1997a, 1997b) observed changes in sperm morphology at the lowest DBP doses, whereas at higher doses sperm morphology, sperm motility (not measured here), and epididymal sperm counts were all adversely affected. Therefore, our results for TOX, although not to be ignored, provide only weak and tentative evidence for an association between exposure to chlorinated water and altered semen quality.

Our analyses of data on other outcomes produced results contrary to our expectation, including those for the relatively new outcomes that measure DNA damage and chromatin maturity in sperm. For example, higher DBP exposures were associated with a higher percentage of normal sperm cells. Also, evaluation of sperm morphology according to the type of defect (i.e., head vs. midpiece vs. tail defects) did not isolate any associations that could potentially be masked by evaluating the whole cell only. In a previous study of exposure to THMs (not considering HAAs or TOX) and semen quality, Fenster et al. (2003) reported an increase in sperm count and concentration with increasing exposure to THM4 and a decrease in percent normal sperm in the highest THM4 exposed group. Our results for THM4 exposure were not consistent with these findings, and our findings for HAAs and TOX could not be compared with theirs because Fenster et al. (2003) did not evaluate these compounds for spermatotoxicity.

The present study had the benefits of a cohort design, a presumed fertile population, data on potential confounders, and modern semen analyses techniques. We had a thorough characterization of water exposures from interview data, including water consumption at home and at work, and data on frequency and duration of bathing and showering. We had the additional benefit of collecting DBP data with greater frequency than in previous epidemiologic studies of DBPs, and in a manner to diminish temporal and spatial variability. The array of DBPs measured included individual species of THMs, all nine HAA species, and TOX. We had a wide range of exposures available to study that resulted from the intentional selection of geographic sites with varying water quality characteristics that produced different distributions of chlorinated and brominated DBP species.

Methodologically, there were a number of important refinements to the study with regard to exposure assessment. The opportunity to consider novel algorithms for estimating exposure via ingestion, inhalation, and dermal routes of exposure allowed for simultaneous exploration of a number of hypothesized pathways by which DBPs could affect male reproductive health.

The largest limitation of the present study is our inability to control for confounding by study site. There was little to no overlap in exposure concentrations between study sites; even after examining many potential confounders, including income and education (surrogates for many other social factors), our strategy of using three different geographic sites to ensure a variety of water quality characteristics created a very strong relationship between residential location and exposure, with the potential for many aspects of site other than water quality to affect sperm quality. That is, if there were unmeasured and even unknown environmental or social influences on sperm quality that vary across our study sites, there would be the potential for those characteristics to confound our measured effects of drinking-water DBPs on sperm quality. To address this concern, we also conducted separate linear correlation analyses with participants from each geographic study site. Although the site-specific analyses have limited power due to lower sample sizes, it is of interest that the puzzling, albeit strong associations between exposure and improved sperm morphology are not significant within a site. These analyses also show no significant linear correlation between exposure and sperm concentration.

The present study had several additional limitations: Our effect estimates were imprecise, and there were few men exposed to concentrations of THM4 above regulatory limits and no men exposed to concentrations of HAA5 above regulatory limits. It is possible that our presumed fertile population accounts for the unusually high sperm counts and low incidence of abnormal sperm counts compared with similar studies enrolling men reporting to infertility clinics or of unknown fertility status. To these means, it could be argued that if our presumed fertile participants were affected by exposure to DBPs so as to lower their sperm counts to a small extent, such an effect might be nondiscernible against the high background sperm count (and its natural variation). We feel this is an unlikely scenario, given the overall higher sperm counts from men in the chlorinated DBP site, an exposed site, compared with those of men in the low DBP site. Even though we measured DBP concentrations weekly, unaccounted for short-term temporal variability may persist (although likely modest in magnitude). Our study was vulnerable to the usual concerns of self-reported information from questionnaire data. Beyond any overall error in reporting, the question of differential error in which men tend to over- or under-report water ingestion, filter use, and duration of bathing and showering is unknown, but seems unlikely. Finally, in generating and interpreting a substantial array of results, the role of random error should be emphasized.

We chose to categorize DBP exposure using percentile cut points rather than regulatory cut points for several reasons. First, we had very few participants exposed to THM4 concentrations above regulatory limits and no participants exposed to HAA5 concentrations above regulatory limits. Further, regulatory limits do not exist for individual species of THMs, HAAs, TOX, THM-Br, or HAA-Br. Finally, the concentration of many of the DBP species we evaluated varied widely by site, making it impossible to select cut points based on DBP concentration that would be useful across study sites. We examined the top 50th, 25th, and 10th percentiles of exposure in the exposed group and found no consistent pattern of association with sperm outcomes and DBP exposures at any of these levels. We chose to present the results from the intermediate exposure level (top 25th vs. bottom 50th percentiles) for the six composite DBP groupings along with partial correlation coefficients that retained the DBP concentrations in their continuous forms.

Previous studies have suggested that exposures to THMs via bathing and showering may be more strongly associated with adverse reproductive outcomes than other exposure indicators (Klotz and Pyrch 1999). Our results did not support these findings.

With the possible exception of sperm concentration and TOX, the results of this study do not support the hypothesis that continuous or intermittent exposure to tap water DBP concentrations within regulatory limits is associated with testicular toxicity as evidence by altered semen quality. Route of exposure (ingestion, inhalation, or dermal) did not change these results. Animal toxicologic studies have demonstrated that haloacetic acids disrupt spermatogenesis; however, these effects are observed at much higher concentrations (Linder et al. 1994a, 1994b, 1995). Confirmation of our results in additional studies of exposure to DBPs in tap water, especially at levels exceeding regulatory limits, would be of value for a more detailed risk characterization.

## Correction

In the “Results” (second paragraph of “Disinfection by-product exposure and sperm outcomes”) of the original manuscript published online, the authors stated that “negative beta coefficients and correlation coefficients indicate decreased adverse semen quality . . .” and “positive beta coefficients and correlation coefficients indicate increased adverse semen quality . . . .” This has been corrected here. Also, in the first paragraph of the “Discussion,” “TOX” has been expanded to “DBPs” in reference to sperm morphology and sperm numbers.

## Figures and Tables

**Table 1:** Selected sociodemographic characteristics for final HMS cohort (based on maternal interview data).

	Eligible men (*n* = 274)	Final (*n* = 228)
Characteristic	No. (%)	No. (%)
Race/ethnicity		
White, non-Hispanic	210 (77.2)	187 (82.0)
Black, non-Hispanic	32 (11.8)	18 (7.9)
Hispanic	6 (2.2)	0 (0.0)
Asian	3 (1.1)	3 (1.3)
Other	21 (7.7)	17 (7.5)
Paternal age (years)		
19–24	38 (14.0)	26 (11.4)
25–29	84 (30.9)	71 (31.1)
30–34	102 (37.5)	89 (39.0)
35–40	48 (17.7)	42 (18.4)
Paternal education		
≤ High school	56 (20.7)	37 (16.3)
Some college	54 (20.0)	42 (18.5)
College graduate plus some graduate work	160 (59.3)	148 (65.2)
Household income (US$/year)		
≤ 20,000	28 (10.4)	19 (8.4)
20,001–40,000	59 (21.9)	43 (19.1)
40,001–80,000	125 (46.5)	111 (49.3)
≥ 80,0001	57 (21.2)	52 (23.1)
Cigarettes/day		
0 (nonsmoker)	224 (83.0)	194 (85.8)
≤ 10 cigarettes/day	23 (8.5)	16 (7.1)
> 10 cigarettes/day	23 (8.5)	16 (7.1)

**Table 2:** Demographic characteristics [no. (%)] of HMS participants by site.

Covariate	All sites (*n* = 228)	Low DBP site (*n* = 91)	Chlorinated DBP site (*n* = 92)	Brominated DBP site (*n* = 45)
Paternal age (years)				
19–24	31 (14)	16 (18)	4 (4)	11 (24)
25–29	77 (34)	36 (40)	28 (30)	13 (29)
30–34	82 (36)	26 (29)	42 (46)	14 (31)
35–40	38 (17)	13 (14)	18 (20)	7 (16)
Race				
Black	18 (8)	9 (10)	6 (7)	3 (7)
Non-black	210 (92)	82 (90)	86 (93)	42 (93)
Ethnicity				
Hispanic	9 (4)	0 (0)	2 (2)	7 (16)
Non-Hispanic	219 (96)	91 (100)	90 (98)	38 (84)
Education				
High school only	35 (15)	18 (20)	3 (3)	14 (31)
Some college	46 (20)	16 (18)	16 (17)	14 (31)
Graduated college	147 (64)	57 (63)	73 (79)	17 (38)
Income (US$/year)				
≤ 40,000	55 (24)	26 (29)	11 (12)	18 (40)
40,001–80,000	109 (48)	41 (45)	48 (52)	20 (44)
≥ 80,001	64 (28)	24 (26)	33 (36)	7 (16)
BMI				
< 18.5 (underweight)	0 (0)	0 (0)	0 (0)	0 (0)
18.5 to < 25 (normal)	63 (28)	23 (25)	30 (33)	10 (22)
25 to < 30 (overweight)	108 (47)	41 (45)	47 (51)	20 (44)
30–< 35 (obese I)	34 (15)	12 (13)	11 (12)	11 (24)
≥ 35 (obese II)	23 (10)	15 (16)	4 (4)	4 (9)
Smoking status				
Yes	93 (41)	37 (41)	32 (35)	24 (53)
No	135 (59)	54 (59)	60 (65)	21 (47)
Alcohol use				
Yes	175 (77)	60 (66)	77 (84)	38 (84)
No	53 (23)	31 (34)	15 (16)	7 (16)
Days abstaining				
2–3	84 (37)	36 (40)	38 (41)	10 (22)
4–8	124 (54)	48 (53)	50 (54)	26 (58)
> 8	20 (9)	7 (8)	4 (4)	9 (20)
Caffeine intake (mg/day)				
None	0 (0)	0 (0)	0 (0)	0 (0)
> 0–150	170 (75)	64 (70)	64 (70)	42 (93)
> 150–300	7 (3)	3 (3)	3 (3)	1 (2)
> 300	6 (3)	2 (2)	3 (3)	1 (2)
Missing	45 (20)	22 (24)	22 (24)	1 (2)
Vitamin use				
Yes	98 (43)	32 (14)	45 (20)	21 (9)
No	130 (57)	59 (26)	47 (21)	24 (11)
Season				
Spring	43 (19)	31 (34)	0 (0)	12 (27)
Summer	60 (26)	17 (19)	22 (24)	21 (47)
Autumn	76 (33)	22 (24)	47 (51)	7 (16)
Winter	49 (21)	21 (23)	23 (25)	5 (11)

**Table 3:** Distribution of outcome variables for all HMS study sites.

Outcome	No.	Mean ± SD	Median (range)
Sperm concentration (millions/mL)	225	114.2 ± 90.1	90.5 (2.4–709.7)
Sperm count (millions/sample)	225	362 ± 311	265 (5–1,845)
Percent normal morphology	228	14.1 ± 5.8	13.3 (2.0–36.0)
Percent abnormal morphology	228	85.9 ± 5.8	86.8 (64.0–98.0)
Percent abnormal head	228	78.6 ± 7.4	79.3 (56.0–97.0)
Percent abnormal midsection	228	22.7 ± 8.9	21.0 (7.0–53.0)
Percent abnormal tail	228	22.2 ± 14.3	18.3 (2.0–65.0)
Percent abnormal cytoplasmic drop	228	1.6 ± 1.5	1.0 (0.0–9.0)
Percent CMA	223	60 ± 20	60 (20–90)
Percent DFI	190	20 ± 10	20 (0–70)

**Table 4:** Results of multivariable^a^ linear regression by semen outcome, with exposure dichotomized as low (bottom 50th percentile) or high (top 25th percentile) for selected groupings of DBPs.

Outcome^b^	THM4 β (95% CL)	HAA5 β (95% CL)	HAA9 β (95% CL)	THM-Br β (95% CL)	HAA-Br β (95% CL)	TOX β (95% CL)
Sperm count^c^	0.35 (−0.02, 0.72)	0.51 (0.08, 0.93)*	0.36 (−0.02, 0.75)	−0.06 (−0.44, 0.33)	0.00 (−0.37, 0.38)	−0.16 (−0.51, 0.19)
Sperm concentration^c^	−0.16 (−0.49, 0.17)	0.06 (−0.27, 0.39)	−0.03 (−0.38, 0.31)	−0.23 (−0.54, 0.07)	−0.20 (−0.51, 0.11)	−0.34 (−0.66, −0.03)*
Percent normal morphology^d^	0.65 (0.34, 0.96)*	0.88 (0.55, 1.21)*	0.67 (0.37, 0.98)*	0.11 (−0.18, 0.40)	0.18 (−0.10, 0.47)	0.04 (−0.24, 0.33)
Percent abnormal head^d^	0.23 (−0.10, 0.57)	0.11 (−0.25, 0.47)	0.28 (−0.06, 0.61)	0.03 (−0.29, 0.34)	0.01 (−0.30, 0.33)	0.20 (−0.12, 0.52)
Percent abnormal midsection^d^	−0.82 (−1.13, −0.51)*	−0.95 (−1.27, −0.64)*	−0.91 (−1.22, −0.59)*	0.15 (−0.16, 0.47)	0.09 (−0.23, 0.41)	0.10 (−0.24, 0.44)
Percent abnormal tail^d^	−1.22 (−1.51, −0.93)*	−1.31 (−1.61, −1.02)*	−1.29 (−1.57, −1.01)*	−0.21 (−0.54, 0.12)	−0.28 (−0.61, 0.04)	−0.28 (−0.60, 0.05)
Percent abnormal cytoplasmic drop^d^	0.33 (0.02, 0.65)*	0.31 (−0.02, 0.64)	0.37 (0.04, 0.70)*	−0.12 (−0.42, 0.18)	−0.10 (−0.41, 0.21)	−0.23 (−0.53, 0.07)
Percent CMA	0.17 (−0.17, 0.50)	−0.04 (−0.38, 0.30)	−0.10 (−0.43, 0.23)	−0.02 (−0.34, 0.31)	−0.01 (−0.34, 0.32)	−0.04 (−0.36, 0.29)
Percent DFI	−0.41 (−0.78, −0.05)*	−0.43 (−0.78, 0.08)	−0.51 (−0.85, −0.17)*	−0.15 (−0.49, 0.18)	−0.18 (−0.52, 0.16)	−0.19 (−0.55, 0.16)

a All models adjusted for age, days abstaining, and education level.

b All outcomes standardized such that SD = variance = 1.00.

c Natural log transformation applied.

d Arc sine transformation applied.

* *p* < 0.05.

**Table 5:** Partial correlation coefficients (r)^a^ for HMS semen outcomes and selected groupings of DBPs on a continuous scale.

Outcome	THM4	HAA5	HAA9	THM-Br	HAA-Br	TOX
Sperm count^b^						
All sites	0.11	0.14	0.110	0.05	0.03	0.08
Low DBP site	−0.04	0.06	−0.01	−0.05	−0.01	0.00
Chlorinated DBP site	−0.17	0.13	0.07	−0.09	−0.06	−0.16
Brominated DBP site	0.13	−0.41*	−0.23	0.19	0.19	0.22
Sperm concentration^b^						
All sites	−0.04	0.01	−0.03	−0.08	−0.08	−0.05
Low DBP site	−0.03	0.03	−0.03	−0.05	−0.04	−0.07
Chlorinated DBP site	−0.28**	0.06	−0.06	−0.22*	−0.22*	−0.16
Brominated DBP site	0.04	−0.07	−0.06	0.09	0.13	−0.14
Percent normal morphology^c^						
All sites	0.24#	0.30#	0.26#	0.08	0.06	0.18**
Low DBP site	0.10	0.10	0.09	0.10	0.09	0.07
Chlorinated DBP site	0.05	−0.03	0.07	0.10	0.08	−0.04
Brominated DBP site	−0.18	−0.12	−0.02	−0.13	0.02	−0.17
Percent abnormal head^c^						
All sites	0.11	0.10	0.11	0.09	0.08	0.10
Low DBP site	0.20	0.10	0.16	0.20	0.15	0.08
Chlorinated DBP site	−0.08	0.10	0.06	−0.14	−0.08	0.16
Brominated DBP site	0.31	0.22	0.28	0.19	0.04	0.22
Percent abnormal midsection^c^						
All sites	−0.25#	−0.33#	−0.25#	−0.03	0.00	−0.15*
Low DBP site	−0.18	−0.15	−0.14	−0.17	−0.13	−0.12
Chlorinated DBP site	0.00	−0.12	−0.14	−0.02	−0.07	−0.02
Brominated DBP site	0.06	0.11	−0.05	−0.02	−0.20	0.35
Percent abnormal tail^c^						
All sites	−0.50#	−0.57#	−0.50#	−0.24#	−0.20**	−0.41#
Low DBP site	−0.14	−0.25*	−0.09	−0.14	−0.08	−0.15
Chlorinated DBP site	0.19	0.20	−0.12	0.18	0.12	−0.09
Brominated DBP site	−0.11	0.15	−0.13	−0.13	−0.22	0.06
Percent abnormal cytoplasmic drop^c^						
All Sites	0.15*	0.20**	0.15*	0.03	0.01	0.13
Low DBP site	−0.02	−0.07	−0.04	−0.02	0.04	0.03
Chlorinated DBP site	−0.25*	−0.08	−0.19	−0.23*	−0.25*	−0.17
Brominated DBP site	0.05	−0.31	−0.11	0.07	0.07	0.15
Percent CMA						
All sites	−0.04	−0.07	−0.08	−0.02	−0.05	−0.10
Low DBP site	−0.02	−0.10	−0.02	0.00	−0.01	−0.05
Chlorinated DBP site	0.29**	−0.03	0.16	0.34**	0.36#	0.27*
Brominated DBP site	0.27	0.07	−0.02	0.29	0.20	−0.23
Percent DFI						
All sites	−0.16*	−0.19**	−0.17*	−0.06	−0.07	−0.16*
Low DBP site	−0.06	0.02	−0.09	−0.09	−0.09	−0.07
Chlorinated DBP site	−0.18	0.06	−0.05	−0.15	−0.15	−0.07
Brominated DBP site	0.28	0.36*	0.29	0.25	0.20	−0.20

a All correlation coefficients were adjusted for age, days abstaining, and education level.

b Natural log transformation applied.

c Arc sine transformation applied.

* *p* < 0.05.

** *p* < 0.01.

# *p* < 0.001.
